# News Media Reports of Respiratory Syncytial Virus in Canada and the United States During the 2022–2023 Respiratory Virus Season: A Cross‐Sectional Study

**DOI:** 10.1002/hsr2.70146

**Published:** 2024-10-30

**Authors:** Sherilyn K. D. Houle, Silvia Luk

**Affiliations:** ^1^ School of Pharmacy University of Waterloo Waterloo Ontario Canada; ^2^ Faculty of Science University of Waterloo Waterloo Ontario Canada

**Keywords:** media, news, older adults, pediatrics, pregnancy, respiratory syncytial virus, vaccination

## 1 Introduction

The 2022/2023 respiratory syncytial virus (RSV) season was marked by early and above‐average case counts, at least partially attributed to the lifting of public health measures such as masking and physical distancing implemented during the early years of the coronavirus disease 2019 (COVID‐19) pandemic [1]. Uniquely, it was also the last season before the availability of vaccines. In May 2023, two recombinant subunit vaccines were approved by the US Food & Drug Administration for use in adults aged 60 years and older (Arexvy and ABRYSVO), with ABRYSVO also indicated for maternal immunization [2, 3].

While hospitalization rates for RSV‐related causes is highest among young children [4, 5], mortality is most marked among older adults [6, 7, 8]. Healthcare professionals also have greater awareness of RSV among pediatric patients than among older adults. In Italy, 21.7% of surveyed general practitioners correctly associated the majority of RSV‐related deaths with older adults, and only 38.9% of participants understood RSV infection as not limited to infants and children [9]. Among US physicians in primary care, 57% reported rarely considering RSV as a potential pathogen in their patients ≥ 50 years old with respiratory disease [10]. Regarding maternal vaccination, a survey of obstetricians and midwives in England reported that 66% of midwives had no or very little awareness of RSV in contrast to 14% of obstetricians, and that routine maternal vaccination against RSV would be recommended by 79% and 89% of midwives and obstetricians, respectively [11]. Research gaps remain related to knowledge and attitudes of other health professionals involved with recommending and administering vaccines (such as nurses and pharmacists) and the public, with one study reporting that 60% of surveyed adults with diabetes in the US had never heard of RSV [12], and 88% of surveyed pregnant women in South England reported having little or no knowledge of RSV [11].

Here, we report on the frequency and content of news media publications related to RSV over the 2022/2023 season in Canada and the United States, hypothesizing that coverage focused on the impact of RSV among the pediatric population with the potential to subsequently contribute to complacency around vaccination of older adults and those who are pregnant.

## 2 Data and Methods

### 2.1 Study Design, Settings, and Data Sources

A cross‐sectional analysis was performed, where websites of news media outlets were searched for print publications including “respiratory syncytial virus” or “RSV” published between October 1, 2022, and May 31, 2023. Outlets were identified from websites that cataloged news media availability in the United States [13] and Canada [14] and were excluded if they did not publish in the English language. Access was purchased for outlets whose publications were available in full only to subscribers.

### 2.2 Eligibility, Screening, and Data Extraction

Publications discussing any aspect of RSV were eligible, including case counts or impact on the health system, pathophysiology, and complications, or vaccine development and approval. Reports were excluded if they were comprised of only a video or audio component without accompanying text. One researcher identified and retrieved eligible publications and performed data extraction of the date of publication, date of access, title, and publication text describing RSV related to the specific populations of pediatrics, older adults, and/or pregnant individuals. Extracted data was reviewed for completion and accuracy by a second researcher with disagreements resolved by discussion and consensus.

RSV case counts over the study period were retrieved from weekly reports by the US Centers for Disease Control and Prevention's National Respiratory and Enteric Virus Surveillance System [15] and the Public Health Agency of Canada's Respiratory Virus Detection Surveillance System [16].

### 2.3 Analysis

Descriptive analyses (frequencies, proportions) were performed to examine RSV case counts over time and the corresponding quantity of publications overall, and those including reports on prespecified subgroups of pediatrics, older adults, and/or pregnant individuals. Analysis was performed using Microsoft Excel for Mac version 16.82.

### 2.4 Ethics

As all data sources are publicly available, research ethics approval was not required.

## 3 Results

Over the study period, 928 unique publications were identified across 29 outlets (Table 1). Of these, 899 (96.9%) discussed RSV among the pediatric population, 310 (33.4%) among older adults, and 104 (11.2%) mentioned maternal immunization. HuffPost was excluded as an eligible outlet as a search function was not available online, and no publications were identified from CNN over the study period. Over this same period, 194,095 positive tests for RSV were reported in the United States and 39,484 in Canada; however, RSV case data was not reported in Canada for Week 51, ending December 24, 2022.

**Table 1:** Included publications and population(s) discussed, by media outlet and country.

Media outlet	Publications (*n*)	Population(s) Discussed (*n*, % of total publications)^a^		
		Pediatrics	Older adults	Pregnancy
United States media outlets				
ABC News	5	5 (100%)	2 (40.0%)	0
Associated Press (AP) News	36	36 (100%)	17 (47.2%)	6 (16.7%)
BBC Online	11	10 (90.9%)	5 (45.5%)	0
Buzzfeed	4	4 (100%)	0	0
CBS News	64	64 (100%)	25 (39.1%)	4 (6.3%)
CNBC	44	37 (84.1%)	32 (72.7%)	11 (25.0%)
Forbes	7	7 (100%)	7 (100%)	2 (28.6%)
Fox News	30	29 (96.7%)	13 (43.3%)	2 (6.7%)
Good Morning America	10	10 (100%)	6 (60.0%)	2 (20.0%)
National Post	50	50 (100%)	6 (12.0%)	4 (8.0%)
NBC News/Today	44	44 (100%)	19 (43.2%)	6 (13.6%)
New York Post	19	18 (94.7%)	9 (47.4%)	4 (21.1%)
People	25	22 (88.0%)	10 (40.0%)	3 (12.0%)
Politico	45	41 (91.1%)	10 (22.2%)	5 (11.1%)
The Hill	75	71 (94.7%)	31 (41.3%)	11 (14.7%)
The New York Times	17	16 (94.1%)	12 (70.6%)	8 (47.1%)
The Sun	26	26 (100%)	4 (15.4%)	0
The Wall Street Journal	19	17 (89.5%)	10 (52.6%)	1 (5.3%)
The Washington Post	25	24 (96.0%)	9 (36.0%)	5 (20.0%)
US News & World Report	56	53 (94.6%)	30 (53.6%)	14 (25.0%)
USA Today	22	22 (100%)	9 (40.9%)	5 (22.7%)
**Overall**	**634**	**606 (95.6%)**	**266 (42.0%)**	**93 (14.7%)**
Canada media outlets				
Aboriginal Peoples Television Network (APTN)	1	1 (100%)	0	0
CBC News	36	35 (97.2%)	17 (47.2%)	3 (8.3%)
CTV News	38	37 (97.4%)	10 (26.3%)	3 (7.9%)
Maclean's	1	1 (100%)	1 (100%)	0
The Globe and Mail	61	61 (100%)	3 (4.9%)	1 (1.6%)
Toronto Star	132	132 (100%)	9 (6.8%)	4 (3.0%)
Toronto Sun	8	8 (100%)	2 (25%)	0
Vancouver Sun	17	17 (100%)	2 (11.8%)	0
**Overall**	**294**	**292 (99.3%)**	**44 (15.0%)**	**11 (3.7%)**

^a^ Sum exceeds total publications as more than one population could be discussed per publication.

**Figure 1:**
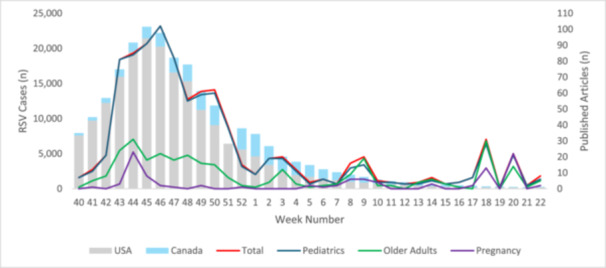
Respiratory syncytial virus cases and publications by week.

Publications and RSV case detections per week are presented graphically (Figure 1) and numerically (Supporting Information: Table S1). Of note, US Food and Drug Administration (FDA) Review Committee recommendations to approve Arexvy and ABRYSVO were released on May 3, 2022 (Week 18) and May 31, 2022 (Week 20), respectively [17, 18]. Publication numbers closely aligned with reported cases, with publications discussing RSV among older adult and maternal populations uncommon early in the season with greater prevalence later in the season and around the time of the FDA committee recommendation to approve Arexvy as the first vaccine available against RSV in the United States.

## 4 Discussion

In the 2022–2023 respiratory virus season, which was marked by an early start and above‐average case counts, news media publications from the United States and Canada on RSV closely aligned in frequency with RSV case counts, with the vast majority of publications discussing RSV among pediatric populations and only one‐third discussing the older adult population despite higher mortality attributable to RSV in the latter. Publications mentioning the older adult population and the potential for maternal immunization spiked in May 2022, aligning with FDA recommendations to approve vaccines targeting these populations; however, these spikes were only one‐third the magnitude of weekly publications discussing the pediatric population earlier in the season. Reasons for such disproportionate reporting are likely complex and establishing these was beyond the scope of this research. Ageism, which is often implicit and subconscious, may take many forms including exclusion from screening or other preventive efforts or therapeutic nihilism [19], where there is lower confidence in an intervention's effectiveness in older adults. Such ageism may result in a societal preference to focus on protecting the young against RSV. Risk aversion related to pregnancy [20] and public perceptions of vaccines as being potentially risky interventions [21] may have also impacted editorial decisions related to the reporting of maternal RSV immunization.

News and media remain trusted sources of health information, even among those expressing vaccine hesitancy. Among 867 residents of Arkansas who were surveyed following receipt of a COVID‐19 vaccine and who expressed hesitancy about vaccination, 16.4% stated that they considered news and social media to be a trusted information source, after healthcare providers (50.2%) and personal contacts (21.8%) [22]. Our finding of low news media reporting on RSV as an infection of concern among older adults has the potential to contribute to complacency around the value of vaccination among this population, who may perceive RSV as a childhood illness. Awareness of maternal vaccination may also be low in the general population with only 11% of news media publications discussing this indication.

While nonprint media (e.g., videos and podcasts) and social media posts by outlets searched in this study were not included, we hypothesize that these are likely to mirror print publications in frequency and content. However, it must be considered that news outlets customize content to their target audience, and demographic characteristics and beliefs among those who consume traditional news media may differ from those who do not [23], potentially biasing our findings and their generalizability. The exclusion of publications in languages other than English may impact generalizability related to awareness and reach among non‐English‐speaking and other minority populations. Finally, we recognize that RSV detections reported to national organizations are an under‐representation of actual prevalence. Future research could consider a parallel examination of nontraditional media sources and the inclusion of reports in non‐English languages to provide a more comprehensive view of information sources consulted by the public.

A biennial pattern of medically attended RSV has been identified using administrative data from Alberta, Canada [24]. This pattern is consistent with the elevated case counts observed in the 2022–2023 respiratory virus season, suggesting another surge season is predicted for the 2024–2025 season when vaccines indicated among both older adults and pregnant individuals will be available. Clinicians are encouraged to proactively offer vaccination to eligible patients and be prepared to discuss the burden of illness among older adults and the benefits of maternal immunization as a preventive strategy for infants, as this research suggests that the volume and comprehensiveness of media reporting and subsequent public awareness may be low. Expansion and ongoing monitoring of news media reporting is encouraged to detect trends that may impact public awareness of immunizations and the diseases they aim to prevent, and to equip clinicians with tools to educate those eligible for RSV immunization on vaccine availability, safety, and effectiveness.

## Author Contributions

**Sherilyn K.D. Houle:** Conceptualization; writing–original draft; methodology; visualization; writing–review & editing; formal analysis; project administration; supervision. **Silvia Luk:** Writing–review & editing; data curation; investigation.

## Conflicts of Interest

Sherilyn K.D. Houle has participated in advisory boards and obtained unrelated research funding from GSK and Pfizer, manufacturers of approved vaccines against RSV. Neither company had any involvement in study design, the collection, analysis, and interpretation of the data, the writing of the report, or the decision to submit the report for publication. Silvia Luk has no conflicts of interest.

## Transparency Statement

The lead author Sherilyn K.D. Houle affirms that this manuscript is an honest, accurate, and transparent account of the study being reported; that no important aspects of the study have been omitted; and that any discrepancies from the study as planned (and, if relevant, registered) have been explained.

## Supporting information

Supporting information.

## Data Availability

Data available upon request to the corresponding author (sherilyn.houle@uwaterloo.ca). The data that support the findings of this study are available from the corresponding author upon reasonable request.
