# Supplementary material for: News Media Reports of Respiratory Syncytial Virus in Canada and the United States During the 2022–2023 Respiratory Virus Season: A Cross‐Sectional Study
Source: Health Sci Rep. 2024 Oct 30;7(11):e70146. doi: 10.1002/hsr2.70146 (PMC11522622; doi:10.1002/hsr2.70146)
Supplement: Supplementary file 1 — Supporting information. [file HSR2-7-e70146-s001.docx]

**SUPPLEMENTARY FILE**

Table S1. Reported RSV cases and publications by calendar week

| **Week Ending** | **Week #** | **RSV Cases Reported** | | **Publications (n)** | **Population Coverage**  **(n, % of total publications of articles)**^†^ | | |
| --- | --- | --- | --- | --- | --- | --- | --- |
|  |  | **USA** | **Canada** |  | **Pediatric** | **Older Adult** | **Pregnancy** |
| 08-Oct-22 | 40 | 7602 | 339 | 7 | 7 (100%) | 1 (14.3%) | 0 |
| 15-Oct-22 | 41 | 9723 | 486 | 12 | 11 (91.7%) | 5 (41.7%) | 1 (8.3%) |
| 22-Oct-22 | 42 | 12233 | 705 | 21 | 21 (100%) | 8 (38.1%) | 0 |
| 29-Oct-22 | 43 | 15972 | 1045 | 81 | 81 (100%) | 24 (29.6%) | 3 (3.7%) |
| 05-Nov-22 | 44 | 19525 | 1309 | 85 | 84 (98.8%) | 31 (36.5%) | 23 (27.1%) |
| 12-Nov-22 | 45 | 21422 | 1661 | 91 | 91 (100%) | 18 (19.8%) | 8 (8.8%) |
| 19-Nov-22 | 46 | 20257 | 1944 | 102 | 102 (100%) | 22 (21.6%) | 2 (2.0%) |
| 26-Nov-22 | 47 | 16580 | 2099 | 82 | 82 (100%) | 18 (22.0%) | 1 (1.2%) |
| 03-Dec-22 | 48 | 15315 | 2392 | 56 | 55 (98.2%) | 21 (37.5%) | 0 |
| 10-Dec-22 | 49 | 11250 | 2683 | 61 | 59 (96.7%) | 16 (26.2%) | 2 (3.3%) |
| 17-Dec-22 | 50 | 9072 | 2795 | 62 | 60 (96.8%) | 15 (24.2%) | 0 |
| 24-Dec-22 | 51 | 6402 | NR | 39 | 38 (97.4%) | 7 (17.9%) | 0 |
| 31-Dec-22 | 52 | 5591 | 3028 | 15 | 14 (93.3%) | 2 (13.3%) | 1 (6.7%) |
| 07-Jan-23 | 1 | 4627 | 3179 | 9 | 9 (100%) | 1 (11.1%) | 0 |
| 14-Jan-23 | 2 | 3407 | 2671 | 19 | 19 (100%) | 4 (21.1%) | 0 |
| 21-Jan-23 | 3 | 2535 | 2043 | 20 | 19 (95.0%) | 12 (60.0%) | 0 |
| 28-Jan-23 | 4 | 1956 | 1885 | 12 | 11 (91.7%) | 3 (25.0%) | 0 |
| 04-Feb-23 | 5 | 1713 | 1667 | 4 | 3 (75.0%) | 1 (25.0%) | 2 (50.0%) |
| 11-Feb-23 | 6 | 1418 | 1361 | 6 | 6 (100%) | 2 (33.3%) | 1 (16.7%) |
| 18-Feb-23 | 7 | 1210 | 1155 | 3 | 3 (100%) | 3 (100%) | 2 (66.7%) |
| 25-Feb-23 | 8 | 1006 | 993 | 16 | 13 (81.3%) | 9 (56.3%) | 6 (37.5%) |
| 04-Mar-23 | 9 | 874 | 821 | 20 | 15 (75.0%) | 19 (95.0%) | 6 (30.0%) |
| 11-Mar-23 | 10 | 719 | 591 | 5 | 4 (80.0%) | 2 (40.0%) | 4 (80.0%) |
| 18-Mar-23 | 11 | 624 | 459 | 4 | 4 (100%) | 2 (50.0%) | 0 |
| 25-Mar-23 | 12 | 538 | 405 | 3 | 3 (100%) | 0 | 0 |
| 01-Apr-23 | 13 | 454 | 313 | 4 | 3 (75.0%) | 4 (100%) | 0 |
| 08-Apr-23 | 14 | 370 | 240 | 7 | 5 (71.4%) | 6 (85.7%) | 3 (42.9%) |
| 15-Apr-23 | 15 | 290 | 278 | 3 | 3 (100%) | 3 (100%) | 0 |
| 22-Apr-23 | 16 | 255 | 253 | 4 | 4 (100%) | 1 (25.0%) | 0 |
| 29-Apr-23 | 17 | 218 | 204 | 7 | 7 (100%) | 0 | 2 (28.6%) |
| 06-May-23 | 18 | 200 | 147 | 31 | 28 (90.3%) | 30 (96.8%) | 13 (41.9%) |
| 13-May-23 | 19 | 200 | 109 | 1 | 1 (100%) | 0 | 0 |
| 20-May-23 | 20 | 181 | 90 | 22 | 21 (95.5%) | 14 (63.6%) | 22 (100%) |
| 27-May-23 | 21 | 160 | 59 | 2 | 2 (100%) | 1 (50.0%) | 0 |
| 03-Jun-23 | 22 | 196 | 75 | 8 | 6 (75.0%) | 5 (62.5%) | 2 (25.0%) |

NR: Not reported

^†^ Sum exceeds total publications as more than one population could be discussed per publication
